# Enhancing the Anticancer and Anti-Inflammatory Properties of Curcumin in Combination with Quercetin, for the Prevention and Treatment of Prostate Cancer

**DOI:** 10.3390/biomedicines11072023

**Published:** 2023-07-18

**Authors:** Michele Pellegrino, Emilia Bevacqua, Luca Frattaruolo, Anna Rita Cappello, Stefano Aquaro, Paola Tucci

**Affiliations:** Department of Pharmacy, Health and Nutritional Sciences, University of Calabria, 87036 Rende, Italy; michele.pellegrino@unical.it (M.P.); emilia.bevacqua@unical.it (E.B.); luca.frattaruolo@unical.it (L.F.); annarita.cappello@unical.it (A.R.C.); stefano.aquaro@unical.it (S.A.)

**Keywords:** tumor, polyphenols, chemoprevention, inflammation, combination therapy

## Abstract

Prostate cancer is the second most common cancer in men. Although epidemiologic studies show that a higher intake of polyphenols, curcumin (CUR), and quercetin (QRT), in particular, result in lower prostate cancer risk, the chemopreventive mechanisms underlying the effects of CUR and QRT have not been fully understood yet, and most investigations were conducted with individual compounds. Here, we investigated the anticancer and anti-inflammatory effects of CUR in combination with QRT, respectively, in a human prostate cancer cell line, PC-3, and in LPS-stimulated RAW 264.7 cells, and found that their combination significantly inhibited proliferation and arrested the cell cycle, inducing apoptosis, so exhibiting synergic activities stronger than single drug use. Moreover, via their antioxidant effects, the combination of CUR and QRT modulated several inflammation-mediated signaling pathways (ROS, nitric oxide, and pro-inflammatory cytokines) thus helping protect cells from undergoing molecular changes that trigger carcinogenesis. Although additional studies, including in vivo experiments and translational studies, are required, this study raises the possibility of their use as a safe, effective, and affordable therapeutic approach to prostate cancer.

## 1. Introduction

Prostate cancer is the most common malignancy diagnosed in men and the second leading cause of cancer death after lung cancer [1]. There is a huge difference in the rate of incidence of prostate cancer between Western (120 per 100,000 in Northern America) and East Asian countries (less than 10 per 100,000 in Asia) [2]. Moreover, when Asian people migrate to Western countries, their prostate cancer incidence rate increases [3]. This supports the idea that lifestyle, aspects of the diet, and environmental factors, as well as genetic factors, promote prostate cancer development. Moreover, recent data have clarified the concept that inflammation is a critical component in tumor progression [4,5]. The relationship between inflammation and cancer is not clear, because many of the molecular and cellular mechanisms mediating this relationship remain unsolved [6]. The current therapies, including radical prostatectomy, chemotherapy, local radiotherapy, and hormonotherapy, are effective in treating localized and androgen-dependent prostate cancer [7,8]. However, these cancerous cells progress to androgen-independent prostate cancer over time, continuously growing despite hormone depletion. At this particular stage, androgen depletion therapy is no longer effective, since these cancerous cells are rendered hormone-insensitive and capable of growing in the absence of androgen. This is a lethal type of disease leading to poor prognosis and is a major contributor to prostate cancer death rates [9,10].

Therefore, novel treatment modalities are needed to treat hormone-resistant tumors and prevent the progression of hormone-sensitive prostate cancer to the hormone-refractory stage. In this view, primary prevention appears as an attractive strategy to decrease prostate cancer incidence by reducing both treatment-related side effects and mortality [11,12].

Among natural compounds, polyphenols represent a large and diverse group that have shown interesting chemopreventive properties without the serious side effects of cytotoxic agents [13]. Natural flavonoids, derived and found abundantly in different plant sources, possess many antioxidant, anti-inflammatory, and anticancer activities by acting through different molecular mechanisms [14]. In addition, they can modulate immune system response and protect normal cells against free radical damage [15]. Several epidemiological studies have shown an inverse relationship between the consumption of plant-based foods, rich in polyphenols, and the incidence of cancer [16]. Among these, curcumin (1,7-bis(hydroxyl-3-methoxyphenyl)-1,6-heptadiene-3,5-dione, CUR), a yellow polyphenolic compound extracted from the rhizome of Curcuma Longa, has been shown to have anticancer properties in a variety of human cancers, both in vitro and in vivo, such as cervical, breast, colon, and lung carcinoma [17,18,19], as well as prostate cancer [20].

Our previous study [21], as well as many works present in the literature, investigated the effects of CUR on different prostate cancer cells, giving key information about the cellular pathways involved in the therapeutic effect [22]. It was found that CUR is effective in significantly reducing the viability of LNCap with a half-maximum inhibitory concentration in the range of 10–20 μM. The evoked mechanism of action involved the Caspase-3 activation and upregulation of nuclear translocation [23], the suppression of anti-apoptotic proteins such as B-cell lymphoma 2 (Bcl-2) and B-cell lymphoma-extra large (Bcl-xL), the upregulation of the poly(ADP-ribose) polymerase cleavage, and the induction of phosphatidylserine translocation to the outer plasma membrane with reduction of membrane integrity [24]. Moreover, the suppression of TNF-induced NF-kB activation [25], the activation of autophagy (upregulation of the microtubule-associated protein 1A/1B-light chain) [26] and a cell-cycle arrest in G1/S [27] or G2/M phase [28], was recorded. More importantly, CUR promoted the production of reactive oxygen species (ROS), inhibited the expression of p110 and p85 subunits of PI3K, and reduced the Akt phosphorylation [23]. It was also proved that CUR was able to enhance the cytotoxic activity of conventional chemotherapeutics, including doxorubicin [21], methotrexate [29], cisplatin, paclitaxel, and docetaxel [30], allowing the dose reduction of the toxic drugs, thereby alleviating their severe side effects.

Similarly, quercetin (3,3′,4′,5,7-pentahydroxyflavone, QRT), a natural flavonoid compound abundant in fruits and vegetables—particularly in onions, apples, red wine, and tea—has demonstrated great chemotherapeutic and chemopreventive effects for prostate cancer through several different mechanisms [31,32]. QRT acts as a pro-apoptotic compound with almost no harmful impacts on normal cells [33]. Its biological action involves the regulation of p21 CDK inhibitor and suppression of pRb phosphorylation, resulting in E2F1 sequestering, with cell cycle arrest at the G1 stage [34]. It should be underlined that QRT affects the activity of several signaling pathways, including the suppression of epithelial to mesenchymal transition [35], inhibition of PI3K, MAPK, and WNT pathways [36], and production of ROS as recorded for many polyphenol compounds [37]. Moreover, both in vitro and in vivo studies have shown that QRT can potentiate the efficacy of concomitant drugs by enhancing their bioavailability and accumulation and by sensitizing cancer cells to these chemotherapeutics [31].

Despite the promising therapeutic value of both CUR and QRT for prostate cancer, their low bioavailability limits their use in clinical practice. Several studies, indeed, showed that only a trace amount of CUR is detected after oral administration [38] due to its low solubility and high transformation rate [39]. CUR, indeed, undergoes phase I and phase II metabolism, with the obtainment of conjugated species showing low biological efficiency and faster excretion [40]. As far as QRT is concerned, it is almost always present in glycosylated form [41] and is absorbed in the small intestine after the removal of sugar units by brush border enzymes [42]. Paradoxically, this process is responsible for a reduction of bioavailability, since the glycosides are generally more bioavailable due to the higher water solubility [43]. Once absorbed, QRT is modified by conjugation within intestinal and hepatic cells and only a small portion enters the bloodstream, while most of the conjugated QRT is excreted back into the intestinal lumen [44].

Although their plasma concentration could be increased by a dietary supplement, they are far from meeting the need. Therefore, to overcome these pharmacokinetic limitations, different solutions have been proposed, including physical modification, chemical modification, and miscellaneous methods [45]. Within the present investigation, we focus on combination therapy, a promising strategy attracting more and more attention in recent years as an effective and efficient strategy compared to monotherapy. The first advantage inherent to the use of combination therapy is that, working in a synergistic or additive manner, it is possible to use a sub-effective dose of the combined therapeutics with considerable reduction of the solubility concerns [46]. Apart from pharmacokinetic benefits, also pharmacodynamic considerations should be taken into consideration. Monotherapy, indeed, hardly leads to the complete inhibition of survival pathways since other alternative pathways may be activated in response to treatment with the development of drug resistance and tumor progression. The use of combination therapy enhances efficacy through more complete inhibition of a major pro-survival pathway or suppression of multiple pro-survival factors, thus reducing drug resistance. Moreover, the lower dose of each drug required for treatment allows the reduction of the side effects related to the administration of the single drug, thus benefiting patients.

To the best of our knowledge, this work is the first example of investigating the chemopreventive effects of CUR and QRT used in combination in a human prostate cancer cell line, PC-3. Additionally, their anti-inflammatory actions were assayed using an experimental model, such as the murine macrophages RAW 264.7.

## 2. Materials and Methods

### 2.1. Cell Cultures and Treatments

The human prostate cancer PC-3 cell line was obtained from American Type Culture Collection (ATCC) and maintained at 37 °C in 5% CO_2_ in a culture medium. PC-3 cells were grown in RPMI 1640 medium supplemented with 10% fetal bovine serum (FBS), 1% L-glutamine, and 1% penicillin/streptomycin. For experiments, cells were plated in complete medium, and, 24 h later, treated in medium with 2% serum for the indicated times, as described in the Results sections.

Murine macrophages RAW 264.7 cell line were purchased from ATCC, UK (No. TIB-71) and cultured in DMEM medium supplemented with 10% FBS, 2 mM L-glutamine, and 1% penicillin/streptomycin in a humidified atmosphere containing 5% CO_2_.

All cell types were cultured at 37 °C and used within 4 months after frozen aliquots resuscitations (less than 20 passages). PC-3 and RAW 264.7 cells were tested monthly for negativity to mycoplasma using the MycoAlert Mycoplasma Detection Kit (Lonza, Gampel-Bratsch, Switzerland). All chemicals were from Sigma/Merck (Darmstadt, Germany).

### 2.2. Preparation of Curcumin and Quercetin Stock Solution

Curcumin (CUR) is a yellow-orange powder, contained in amber packaging because it is photosensitive and stored, according to the manufacturer’s instructions, at −20 °C. Quercetin (QRT) is a yellow powder, chemically stable under standard environmental conditions and stored, according to the manufacturer’s instructions, at +4 °C. A 4 mM stock solution of CUR or QRT was prepared in dimethyl sulfoxide (DMSO), and serial dilutions of the stock solution were prepared and combined at the time of use and then used to treat the cells. These solutions were diluted in the RPMI 1640 culture medium without serum to reach the desired final concentration (0.5, 1, 5, 10, 20 μM). The final concentration of DMSO in the cell cultures was equal to or less than 0.5% and was not toxic to the cells. All chemicals, including CUR and QRT, were from Sigma/Merck (Darmstadt, Germany).

### 2.3. Cell Viability Assay

Cell viability was assessed by quantifying the mitochondrial-dependent reduction of 3-(4,5-dimethyl-2-thiazolyl)-2,5-diphenyl-2H-tetrazolium bromide (MTT) to formazan by living cells. Briefly, PC-3 cells were seeded at a density of 2 × 10^4^ cells/well in 48-well plates (Corning Inc. Tewksbury, MA, USA) and incubated overnight in a complete medium. After 24 h, media were aspirated and replaced with fresh RPMI with 2% serum, and cells were exposed to different concentrations (5, 10, 20 μM) of CUR or QRT, or in combination, for 24 and 48 h. After the exposure treatment, MTT solution was added to each well (to a final concentration of 0.5 mg/mL) and plates were incubated at 37 °C for 2 h until the formation of formazan crystals. Then, the medium was removed, and 200 μL of DMSO was added to the formazan precipitate in each well. The absorbance (Abs) of the samples was measured at 570 nm using a microplate reader (Synergy H1, BioTek Inc., Winooski, VT, USA). The optical density (OD) was calculated as the difference between the absorbance at the reference wavelength and that at the test wavelength. Percent viability was calculated according to the following equation:

(1) $$Viability\left(\%\right)=\frac{{OD}_{sample}}{{OD}_{control}}\times 100$$

All chemicals were from Sigma/Merck (Darmstadt, Germany).

### 2.4. Cell Cycle Analysis

To assess cell cycle analysis, PC-3 cells were seeded in 6-well plates, at a density of 2.5 × 10^5^ cells/well, and treated with vehicle (DMSO) or 10 μM of CUR or QRT, alone or in combination, for 24 h. Next, PC-3 cells were harvested after treatment by trypsinization, rinsed twice with PBS, pelleted by centrifugation, and fixed in 70% cold ethanol for 30 min at 4 °C. Then, samples were washed with PBS and stained using a solution containing 3.8 mM sodium citrate, 100 μg/mL RNAse, 50 μg/mL propidium iodide (PI), and 0.1% Igepal in PBS for 1 h at 37 °C. The samples were then analyzed using a CytoFLEX Beckman flow cytometer (Beckman Coulter Inc., Brea, CA, USA). All chemicals were from Sigma/Merck (Darmstadt, Germany).

### 2.5. Reactive Oxygen Species (ROS) Production Assay

The production of ROS in cells was detected by using the general oxidative stress indicator (CM-H_2_DCFDA, Invitrogen, Carlsbad, CA, USA). Then, 1 × 10^5^ PC-3 cells/well were seeded in 6-well plates and treated with DMSO or 10 μM of CUR, or 10 μM of QRT, or in combination, for 24 h. Cells treated with 1 mM H_2_O_2_ for 4 h were used as the positive control. The next steps were also performed in the dark, to avoid probe oxidation. After treatment, cells were collected, and the pellet was incubated in a mix containing PBS and 5 μM of the dye chloromethyl-2′,7′-dichlorofluorescien diacetate (CM-H_2_DCFDA) at 37 °C with 5% of CO_2_. After 1 h, cells were incubated with a growth medium at 37 °C for 20 min. Finally, the fluorescence of samples was quantified with a fluorimeter (Synergy H1 microplate reader, BioTeck, Winooski, VT, USA) in an excitation/emission range of 492–495/517–527 nm. In each sample, fluorescence intensity was normalized on viable cells and counted using a 10× objective on a microscope (CKX31 Olympus). Unless otherwise indicated, all chemicals were from Sigma/Merck (Darmstadt, Germany).

### 2.6. Tunel Assay

Apoptosis was tested by enzymatic labeling of DNA strand breaks using terminal deoxynucleotidyl transferase-mediated deoxyuridine triphosphate nick end-labeling (TUNEL) assay, following the guidelines of the manufacturer (TUNEL assay kit, Promega, Madison, WI, USA), according to Armentano et al. [47]. Briefly, PC-3 cells were treated with vehicle alone (DMSO) or 10 μM of CUR, 10 μM of QRT, or combined treatment, for 48 h. Then, nuclear staining was achieved using 0.2 mg/mL 4′,6-diamidino-2-phenylindole (DAPI), and apoptotic nuclei were visualized using a fluorescent microscope (Olympus BX4 equipped with CSV1.14 software employing a CAMXC-30 for image acquisition). Unless otherwise indicated, all chemicals were from Sigma/Merck (Darmstadt, Germany).

### 2.7. Inhibition of Nitric Oxide (NO) Production in Lipopolysaccharide (LPS)-Stimulated RAW 264.7 Cells

RAW 264.7 cells were seeded at a density of 2 × 10^5^ cells/well in 24 plates and incubated overnight in a complete medium. After 24 h, media were replaced with a mix of fresh DMEM and LPS (1 μg/mL) and vehicle control (DMSO) or different concentrations of CUR or QRT, alone or in combined treatment. After treatment, the presence of nitrite (a stable oxidized product of NO) was measured in cell culture media by using the Griess reagent, as previously reported [48]. Briefly, 100 μL of cell culture supernatant was incubated with 100 μL of Griess reagent at room temperature for 10 min in a 96-well plate, and then the absorbance was measured at 550 nm using a microplate reader (Synergy H1 microplate reader, BioTek). All chemicals were from Sigma/Merck (Darmstadt, Germany).

### 2.8. RNA Extraction, cDNA Synthesis, and qRT-PCR

RAW 264.7 cells were grown in 10 cm dishes to 70–80% confluence and exposed for 6 or 24 h to the vehicle (DMSO), to LPS (1 μg/mL) alone, or in the presence of CUR, QRT, or CUR + QRT 10 μM. The gene expression analyses were carried out as already described [49]. Briefly, total cellular RNA was isolated from RAW 264.7 cells using TRIZOL reagent (Invitrogen) according to the manufacturer’s instructions. RNA samples were reverse transcribed using the OneScript Plus cDNA Synthesis Kit and oligo (dT) primer (ABM) (ThermoFisher Scientific, Waltham, MA, USA). qRT-PCR was performed using an ABI PRISM 7000 Sequence Detection System with SYBR Green Universal PCR Master Mix (Applied Biosystem, ThermoFisher Scientific, Waltham, MA, USA) according to the manufacturer’s recommendations. Assays were performed in triplicate, and the results were normalized using Glyceraldehyde 3-phosphate dehydrogenase (GAPDH) mRNA levels. Relative mRNA levels were calculated using the ΔΔCt method compared to the control group. All the primers used for amplifications are listed below:

IL-6:

- Fw: CTGCAAGAGACTTCCATCCAG
- Rv: AGTGGTATAGACAGGTCTGTTGG

IL-1β:

- Fw: GAAATGCCACCTTTTGACAGTG
- Rv: TGGATGCTCTCATCAGGACAG

TNF-α:

- Fw: CAGGCGGTGCCTATGTCTC
- Rv: CGATCACCCCGAAGTTCAGTAG

GAPDH

- Fw: ACCACAGTCCATGCCATCAC
- Rv: TCCACCACCCTGTTGCTGTA

### 2.9. Statistical Analysis

Data were reported as mean values ± standard deviation (SD) of three independent experiments. One-way or two-way analysis of variance (ANOVA), followed by Dunnett’s method, was used to generate statistical analyses. Tukey’s post-hoc test was used to study differences between groups using the GraphPad Prism 8.0 (GraphPad Software, Inc., La Jolla, CA, USA) software. *p* values ≤ 0.01 were considered statistically significant.

## 3. Results and Discussion

### 3.1. Curcumin in Combination with Quercetin Increases its Antiproliferative Effects in Prostate Cancer Cell Line

**Figure 1:**
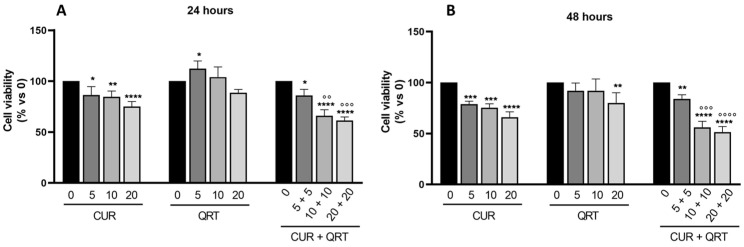
Cell viability was assessed by MTT assay in PC-3 prostate cancer cells. PC-3 were treated for 24 (**A**) or 48 (**B**) hours with 5, 10, or 20 µM CUR or QRT, alone or in combination. Data were reported as mean values ± SD of three independent experiments. Two-way ANOVA followed by Dunnett’s method: * *p* < 0.01, ** *p* < 0.001, *** *p* < 0.0001, **** *p* < 0.00001 vs. control; °° *p* < 0.001, °°° *p* < 0.0001, °°°° *p* < 0.00001 vs. CUR or QRT single treatments.

The effects on cancer cell proliferation of CUR or QRT were evaluated in the PC-3 prostate cancer cell line by using the (3-(4,5-dimethylthiazol-2-yl)-2,5 diphenyl tetrazolium bromide) MTT assay. For this purpose, the PC-3 cell line was treated with increasing concentrations (5, 10, 15, 20 µM) of CUR or QRT or combination treatment (CUR + QRT) for 24 and 48 h, as shown in Figure 1A,B respectively.

The results showed that single treatments (CUR or QRT) exerted a reduction in PC-3 cell proliferation in a concentration and time-dependent manner. Specifically, the combined treatment (CUR + QRT) showed a reduction in cell proliferation compared to QRT individual treatments by almost 15%, at a low concentration (5 µM) and after 24 h (Figure 1A), but this reduction can be traced back to the action of CUR. The reduction of cell viability was already significant at the lowest concentration until reaching a reduction in cell proliferation of almost 35% with 10 µM CUR + QRT after 24 h of treatment, compared to the control cells. This result highlights that the combination treatment is more effective than the single treatment.

On the other hand, by increasing the concentration of the combination treatment up to 20 µM, there was just a slight additional decrease in cell viability (about 5%).

Furthermore, by analyzing Figure 1B, it stands out that the combination treatment does not exert a time-dependent increase in cytotoxicity. A similar trend was observed over 24 h. For these reasons, we used for further experiments a 10 µM concentration and an incubation time of no longer than 24 h.

### 3.2. Curcumin and Quercetin Induce Cell Cycle Arrest

To understand if the antiproliferative effect of CUR + QRT could be a consequence of cell cycle perturbations, we assessed cell cycle analysis by flow cytometry and estimated the deployment of cells within the three main phases of the cell cycle by propidium iodide (PI) staining. The PC-3 cell line was treated for 24 h with the vehicle (DMSO) or with the compounds CUR or QRT, alone or in combination treatment, at 10 µM.

**Figure 2:**
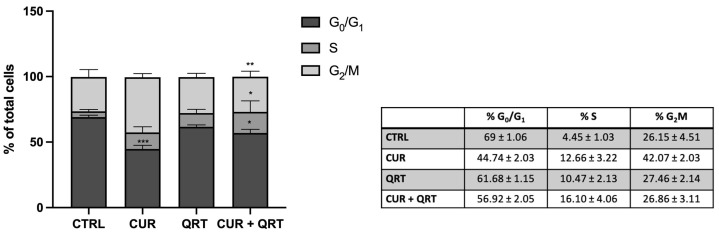
Cell cycle distribution by flow cytometry of PC-3 cells treated with 10 µM CUR, 10 µM QRT, or combination treatment for 24 h. CUR or QRT alone showed an increase in cell percentage in the S phase. The combination treatment showed a higher increase in the S phase compared with CUR or QRT alone. Data were reported as mean values ± SD of three independent experiments. Two-way ANOVA followed by Dunnett’s method: * *p* < 0.01, ** *p* < 0.001, *** *p* < 0.0001 vs. control.

Our results revealed that CUR arrested cells in the G2/M phase, while QRT arrested PC-3 cells in the S phase. The combination treatment (CUR + QRT) showed a significant increase in cells in the S phase compared to QRT (Figure 2).

In particular, after 24 h, we observed a significant increase in the percentage of PC-3 cells in the G2/M phase (+16%) treated with CUR, while the treatment with QRT showed an increase in the percentage of PC-3 cells in the S phase (+6%) but no significant increase was revealed in the G2/M phase. Conversely, the treatment with CUR + QRT showed a significant increase in the S phase (+12%), more evident than QRT alone.

### 3.3. Curcumin and Quercetin Induce Cell Death by Activation of Apoptotic Pathways

It is known that CUR induces cell death through apoptotic processes, a programmed cell death responsible for removing damaged or abnormal cells by ensuring tissue integrity and function in the cellular system. Thus, we wondered if the combination treatment with QRT was able to enhance the pro-apoptotic activity of CUR and we performed a TUNEL assay in order to assess DNA fragmentation, which is a late event of apoptosis. To this end, PC-3 cells were incubated for 48 h with 10 µM of CUR or QRT or with the combination treatment.

**Figure 3:**
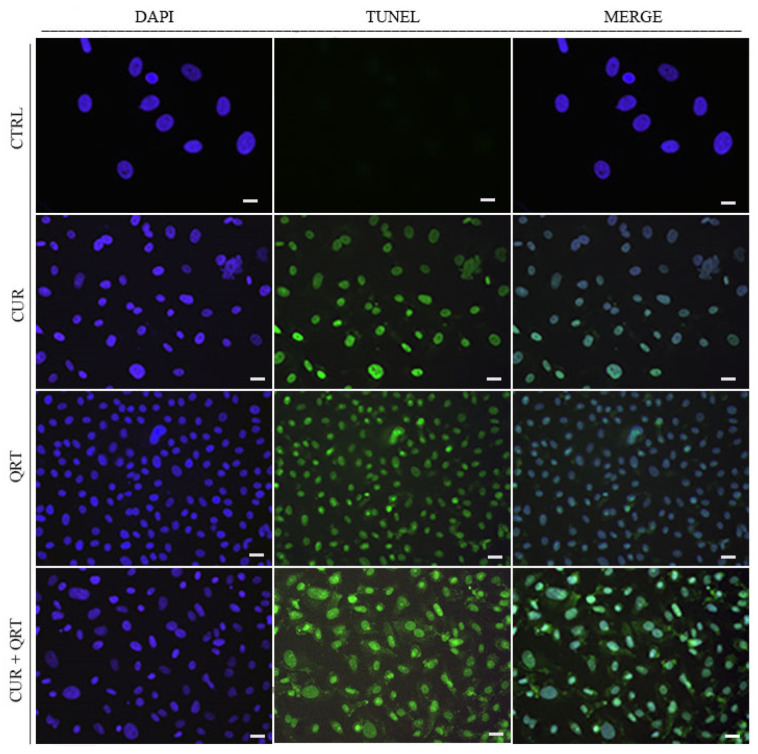
TdT-mediated dUTP nick end-labeling (TUNEL) assay in PC-3 cells treated for 48 h with vehicle alone (CTRL) or 10 µM of CUR or QRT, alone or in combination (CUR + QRT). DAPI was used for DNA staining (scale bar: 50 μm, magnification 20×).

Our results showed an increased number of TUNEL-positive cells (Figure 3) when PC-3 cells were treated with the co-treatment CUR + QRT compared to the control cells (CTRL) or to the single treatments (CUR or QRT).

### 3.4. The Combination Treatment Induces ROS Levels

**Figure 4:**
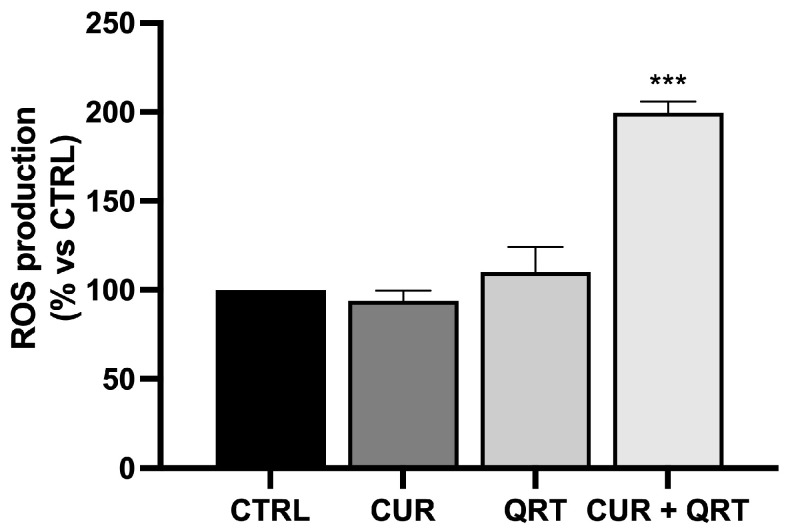
ROS levels production (expressed as % of ROS production compared to the control) was assayed using the CM-H_2_DCFDA dye in PC-3 cells after 24 h of treatment with 10 µM CUR or QRT or their combination treatment. Data were reported as mean values ± SD of three independent experiments. Two-way ANOVA followed by Dunnett’s method: *** *p* < 0.0001 vs. control.

Natural compounds, including CUR and QRT, via their antioxidant property, play a key role in cancer chemoprevention by suppressing oxidative stress-induced DNA damage [50]. In addition, they modulate several oxidative stress-mediated signaling pathways through their antioxidant effects and, ultimately, protect cells from undergoing molecular changes that trigger carcinogenesis. In several instances, however, the pro-oxidant property of these compounds has been observed for cancer treatment [51]. Further, in vitro and in vivo studies show that several phytochemicals potentiate the efficacy of chemotherapeutic agents by exacerbating oxidative stress in cancer cells [52]. Therefore, we investigated the role of the CUR and QRT, used in combination, in regulating oxidative stress in the context of cancer chemoprevention and treatment. To investigate the level of reactive oxygen species (ROS) induced by CUR, QRT, or combined CUR + QRT, we used CM-H_2_DCFDA, a cell-permeable fluorogenic probe that is deacetylated by intracellular esterases and oxidized in the presence of ROS, becoming highly fluorescent. As we expected (Figure 4), the PC-3 cell death was associated with an increase in intracellular ROS production.

In particular, PC-3 cells were treated with the vehicle (DMSO) or 10 µM of CUR, QRT, or CUR + QRT for 24 h. Our results demonstrated no significant modulation in ROS levels when PC-3 cells were treated with CUR or QRT alone, while a different behavior was observed in cells treated with 10 µM CUR + QRT. Indeed, the combination treatment led to an increase of the ROS levels by up to 200% compared to control cells.

However, polyphenols are known to exert apoptotic effects by inducing oxidative stress at high concentrations [50]. Our results have shown that, at lower concentrations, CUR or QRT individually were not able to regulate intracellular ROS function as signaling molecules or triggers to initiate downstream events in regulating cell viability, cell cycle, and apoptosis. On the other hand, the combination of CUR and QRT at the same concentration led to significant changes. This is in agreement with the well-known antioxidant/pro-oxidant paradox [53]: the main effect by which polyphenol molecules exert their antioxidant effect is, indeed, related to their ability to quench free radicals by a redox reaction, with stable polyphenol radicals being formed as a product of the reaction. At high concentrations of either the ROS or polyphenol species, their intrinsic redox activity led to the insurgence of an uncontrolled redox reaction, further promoting an enhancement in ROS formation. It should be underlined that we explored ROS production upon treatment with 10 μM CUR + QRT since the results of viability experiments suggested this value as a cutting point between antioxidant (no significant modification of viability) and pro-oxidant effect (significant improvement of toxicity).

### 3.5. Effects of Curcumin and Quercetin on Nitric Oxide Production in LPS-Activated RAW 264.7 Macrophages

Inflammation is an essential protective process that preserves the integrity of organisms against physical, chemical, and infectious insults. These events can lead to a state defined as “initiation”, which involves DNA alterations and irreversible damage that can persist until the occurrence of a second type of stimulation, called “promotion” [54]. The different promoters, directly or indirectly, can induce cell proliferation and recruit inflammatory cells. Furthermore, the systemic inflammatory response is associated with the production of ROS, nitric oxide (NO), and cytokines such as tumor necrosis factor (TNF-α) [55]. However, an inflammatory response to multiple insults often mistakenly leads to damage to normal tissues. At first, we evaluated the effect of the combination treatment in reducing NO production, since it is well known that NO, a gaseous, free radical, short-lived molecule, possesses multiple functions in a variety of biological phenomena: it is believed to induce vasodilatation in the cardiovascular system and is involved in immune responses by cytokine-activated macrophages [56]. Inflammation is the major driver implicated in the pathogenesis of many diseases, including cancer. Several inflammatory stimuli, such as lipopolysaccharide (LPS), can promote many chronic inflammatory diseases [57]. Many plants used in traditional medicines for rejuvenation therapy and chronic disorders have been shown to stimulate immune responses, and several active principles have been isolated from plants [58].

The anti-inflammatory potential of CUR and QRT was investigated by monitoring their ability to modulate the production of NO in LPS-stimulated RAW 264.7 cells, murine macrophages widely used as an in vitro model to study inflammatory pathways. NO production was measured in terms of the amount of nitrite, its stable product [59].

**Figure 5:**
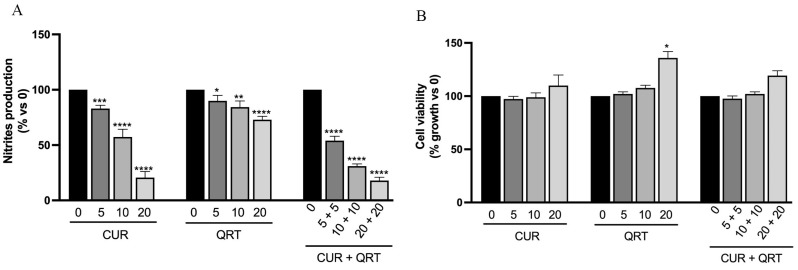
(**A**) Nitrites production assessment in LPS (Lipopolysaccharide) stimulated RAW 264.7 cells after treatment with different concentrations of CUR, QRT, or their combined treatment, as indicated. Results were quantified by Griess Assay; (**B**) Cell growth assessment of RAW 264.7 after treatment for 24 h with CUR, QRT, or combination treatment. The cell viability assessment was defined by MTT Assay. Data were reported as mean values ± SD of three independent experiments. Two-way ANOVA followed by Sidak’s method: * *p* < 0.01, ** *p* < 0.001, *** *p* < 0.0001, **** *p* < 0.00001, vs. control.

In particular, treatment with different concentrations of CUR induced a significant dose-dependent reduction after 24 h, while the anti-inflammatory activity of QRT was not that obvious at the same concentrations. An interesting behavior was observed in macrophages treated with a blend of CUR + QRT, which, already at the lowest concentrations (5 μM), displayed an evident reduction in the nitrites production of about 50% compared to control, but also to individual treatments (Figure 5A). We observed a dose-dependent reduction after combined treatment compared with the single treatment.

To ensure that the concentration of CUR or QRT used did not cause cell damage and to demonstrate that reduced nitrites production was not associated with cell death, the result was normalized using the MTT assay (Figure 5B). Hence, in the presence of LPS, there were no adverse effects on the growth of RAW 264.7 macrophages at a concentration of up to 20 μM CUR and QRT.

### 3.6. Curcumin and Quercetin Modulate Pro-Inflammatory Cytokines

In addition to NO, inflammation appears to be driven by inflammatory cytokines such as IL-6, IL-1β, and TNF-α [60]. Hence, there is a strong interest in agents that can block the generation or actions of inflammatory cytokines. Cytokines are important mediator proteins, essential in networking communication for the immune system, and can be produced by lymphocytes (lymphokines) or monocytes (monokines) with pro-inflammatory and anti-inflammatory effects. The effects of polyphenols on various inflammatory processes and immune functions have been extensively reviewed in the literature, and it has been demonstrated that they may induce a considerable reduction in the generation of inflammatory cytokines via multiple mechanisms, including the inhibition of several enzymes activated in certain inflammatory conditions [61,62].

**Figure 6:**
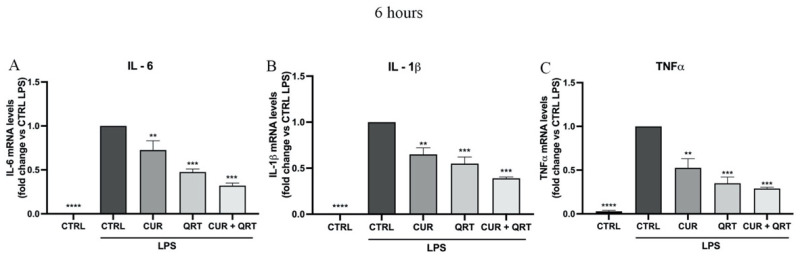
Effects of treatments on the mRNA expression levels of pro-inflammatory cytokines: (**A**) Interleukin-6 (IL-6), (**B**) Interleukin-1 beta (IL-1β), and (**C**) Tumor Necrosis Factor-alpha (TNFα). RAW 264.7 cells, murine macrophages, were stimulated with lipopolysaccharide (LPS) and treated with 10 µM CUR, 10 µM QRT, or with the combined treatment for 6 h. Data were reported as mean values ± SD of three independent experiments. Two-way ANOVA followed by Tukey’s method: ** *p* < 0.001, *** *p* < 0.0001, **** *p* < 0.00001 vs. control.

In this study, the mRNA expression levels of IL-6, IL-1β, and TNF-α were measured, as shown in Figure 6, after LPS stimulation and simultaneous treatment with CUR, QRT, or a combination of them. Therefore, to investigate the ability of CUR or QRT to reduce the expression of pro-inflammatory cytokines, RAW 264.7 cells were treated with 10 μM of CUR or QRT, alone or in combination, for 6 h. Since the production of nitrites was observed after 24 h, the levels of proinflammatory cytokines should have been reduced in a shorter time, as they are produced before nitrites because NO is a molecular mediator that is increasingly implicated in cytokine action. For this reason, we measured the cytokines after 6 h of treatment, and the result is shown in Figure 6.

In particular, the co-treatment of CUR + QRT showed at the transcriptional level a significant reduction of the expression of IL-6, IL-1β, and TNF-α (Figure 6A–C) compared to the control cells stimulated with LPS (CTRL LPS) but also to the single treatments (CUR or QRT). As pro-inflammatory cytokines, TNF-α, IL-6, and IL-1β are involved in the initiation and propagation of the pro-inflammatory cascades, recruiting and activating various inflammatory cells for recovery purposes, and their over-production leads to chronic inflammation and cell apoptosis. The synergistic effects detected in RAW 264.7 cells exposed to the combination of CUR and QRT allow us to identify the blend of the two flavonoids as an effective inhibitor of cytokine cascade, with a strong anti-inflammatory potential. CUR and QRT, indeed, may exert their anti-inflammatory and immunomodulatory properties by suppressing the activation of ERK and p38 MAP kinase and NF-kappaB signal transduction pathways [63,64,65].

The strong anti-inflammatory potential, together with the synergistic anticancer activity of the two compounds, allowed for hypothesizing their effective use for prostate cancer treatment. This statement finds confirmation in the literature data showing that the co-administration of polyphenols and conventional chemotherapeutics can significantly enhance the anticancer activity while reducing the side effects [66]. Thus, future in vitro and in vivo experiments will focus on the development of co-treatment involving the use of combined CUR + QRT with cytotoxic drugs such as doxorubicin and cisplatin to confirm the expectations that the three-compound combination is more effective than the drug plus single polyphenol treatments.

## 4. Conclusions

Our work showed that combinations of CUR and QRT were more effective in inhibiting cancer growth and inflammation than treatment with a single compound. The combination, through the reciprocal interactions of the two flavonoids, including synergy, may modulate the bioavailability of single natural compounds and exercise pleiotropic effects by simultaneously affecting multiple metabolic pathways involved in carcinogenesis. Moreover, this proper combination enables the use of lower doses of individual components without compromising their efficacy and opens up the possibility of developing more effective strategies against various human pathologies, including cancer.

Further experiments are being performed to underline the biological and molecular mechanisms behind the enhanced antiproliferative effects of the combination treatment, including the possibility to reverse the multi-drug resistance mechanisms and the determination of the pharmacokinetic profiles in vivo. This could further improve the clinical outcome of the preliminary data here reported. Although additional studies, including in vivo experiments or translational studies, are required to further confirm the therapeutic and preventive effect of using CUR and QRT in combination on human prostate cancer, the above results indicate the potential of CUR in combination with QRT, in diet or as treatment, as an alternative and non-toxic modality for prostate cancer prevention, treatment, or co-treatment with conventional therapy, by which the clinician may prevent the progression of prostate cancer to its hormone refractory state or treat advanced prostate cancer.

Moreover, this novel approach represents a rationale for the treatment of this deadly disease that will not only help to decline the incidence rate and improve the adverse effects of prostate cancer treatment but also offer more benefits to patients and alleviate the great burden for both the state and prostate cancer patients.

## Data Availability

Not applicable.
